# Epidemiologic Investigation of Immune-Mediated Polyradiculoneuropathy among Abattoir Workers Exposed to Porcine Brain

**DOI:** 10.1371/journal.pone.0009782

**Published:** 2010-03-19

**Authors:** Stacy M. Holzbauer, Aaron S. DeVries, James J. Sejvar, Christine H. Lees, Jennifer Adjemian, Jennifer H. McQuiston, Carlota Medus, Catherine A. Lexau, Julie R. Harris, Sergio E. Recuenco, Ermias D. Belay, James F. Howell, Bryan F. Buss, Mady Hornig, John D. Gibbins, Scott E. Brueck, Kirk E. Smith, Richard N. Danila, W. Ian Lipkin, Daniel H. Lachance, P. James. B. Dyck, Ruth Lynfield

**Affiliations:** 1 Infectious Disease, Epidemiology, Prevention and Control Division, Minnesota Department of Health, Saint Paul, Minnesota, United States of America; 2 Epidemic Intelligence Service, Office of Workforce and Career Development, Centers for Disease Control and Prevention, Atlanta, Georgia, United States of America; 3 Division of Viral and Rickettsial Diseases, National Center for Zoonotic, Vector-borne and Enteric Diseases, Centers for Disease Control and Prevention, Atlanta, Georgia, United States of America; 4 Public Health Preparedness and Emergency Response, Indiana State Department of Health, Indianapolis, Indiana, United States of America; 5 Division of Public Health, Nebraska Department of Health and Human Services, Lincoln, Nebraska, United States of America; 6 Center for Infection and Immunity, Columbia University, New York, New York, United States of America; 7 Division of Surveillance, Hazard Evaluations, and Field Studies, National Institute for Occupational Safety and Health, Cincinnati, Ohio, United States of America; 8 Department of Neurology, Mayo Clinic, Rochester, Minnesota, United States of America; Finnish Institute of Occupational Health, Finland

## Abstract

**Background:**

In October 2007, a cluster of patients experiencing a novel polyradiculoneuropathy was identified at a pork abattoir (Plant A). Patients worked in the primary carcass processing area (warm room); the majority processed severed heads (head-table). An investigation was initiated to determine risk factors for illness.

**Methods and Results:**

Symptoms of the reported patients were unlike previously described occupational associated illnesses. A case-control study was conducted at Plant A. A case was defined as evidence of symptoms of peripheral neuropathy and compatible electrodiagnostic testing in a pork abattoir worker. Two control groups were used - randomly selected non-ill warm-room workers (n = 49), and all non-ill head-table workers (n = 56). Consenting cases and controls were interviewed and blood and throat swabs were collected. The 26 largest U.S. pork abattoirs were surveyed to identify additional cases. Fifteen cases were identified at Plant A; illness onsets occurred during May 2004–November 2007. Median age was 32 years (range, 21–55 years). Cases were more likely than warm-room controls to have ever worked at the head-table (adjusted odds ratio [AOR], 6.6; 95% confidence interval [CI], 1.6–26.7), removed brains or removed muscle from the backs of heads (AOR, 10.3; 95% CI, 1.5–68.5), and worked within 0–10 feet of the brain removal operation (AOR, 9.9; 95% CI, 1.2–80.0). Associations remained when comparing head-table cases and head-table controls. Workers removed brains by using compressed air that liquefied brain and generated aerosolized droplets, exposing themselves and nearby workers. Eight additional cases were identified in the only two other abattoirs using this technique. The three abattoirs that used this technique have stopped brain removal, and no new cases have been reported after 24 months of follow up. Cases compared to controls had higher median interferon-gamma (IFNγ) levels (21.7 pg/ml; vs 14.8 pg/ml, P<0.001).

**Discussion:**

This novel polyradiculoneuropathy was associated with removing porcine brains with compressed air. An autoimmune mechanism is supported by higher levels of IFNγ in cases than in controls consistent with other immune mediated illnesses occurring in association with neural tissue exposure. Abattoirs should not use compressed air to remove brains and should avoid procedures that aerosolize CNS tissue. This outbreak highlights the potential for respiratory or mucosal exposure to cause an immune-mediated illness in an occupational setting.

## Introduction

In October 2007, the Minnesota Department of Health (MDH) was notified of 10 patients experiencing unusual neurologic illness who worked at a swine abattoir (Plant A) in southeastern Minnesota. It was reported that patients experienced significant sensory symptoms including numbness and tingling as well as limb weakness consistent with polyradiculoneuropathy, and was initially referred to as progressive inflammatory neuropathy (PIN)[1] now described as sensory predominant, immune-mediated polyradiculoneuropathy (IP). Among those that had been evaluated in a health care setting, cerebrospinal fluid (CSF) protein was elevated in the absence of pleocytosis and several had evidence of spinal nerve root or spinal cord inflammation on Magnetic Resonance Imaging (MRI). The illness appeared to be associated with working in plant A and was unlike previously described occupational associated illnesses.

Plant A employed 1,300 workers and slaughters >19,000 hogs per day. The patients worked in the warm-room, the area where hogs are eviscerated and initially processed. Seven patients worked at the head-table, the area within the warm-room where skin, skeletal muscle, and brain are removed from severed swine heads. Two shifts, each employing 200 workers, operate in the warm-room, with 35–40 workers at the head-table during each shift.

Considering the unusual nature of the illness and apparent clustering, a public health response and epidemiologic investigation was initiated.

## Methods

### Initial Case Assessment and Occupational Investigation

The public health investigation was conducted by the MDH under the authority provided under Minnesota Statute 144.05[2] and State Rule 4605.7050[3]. As such, this investigation was considered to be a public health response and Institutional Review Board review and approval was not required. We reviewed all available medical records for reported Minnesota patients. A site visit of Plant A was conducted and work practices, slaughtering techniques, and safety equipment were examined. We reviewed material safety data sheets regarding all chemicals used, ventilation blueprints, and maintenance records. We reviewed occupational health and Workers' Compensation records to supplement case-finding. Potential cases were interviewed using a detailed standard questionnaire assessing neurologic and infectious symptoms within the past 12 months, work history, known risk factors for inflammatory neuropathies, personal health history, family history, and potential exposures.

### Case Definition

**Figure 1:**
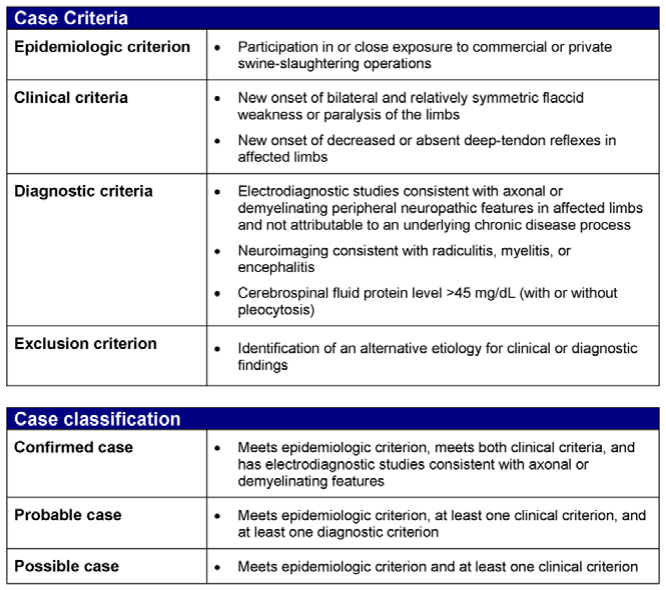
Case definition of immune-mediated polyradiculoneuropathy[1].

On the basis of initial medical record findings and interview data, a stratified case definition, including epidemiologic, clinical, and diagnostic criteria, was developed (Figure 1)[1]. The purpose of the development of this case definition was to identify risk factors associated with illness. Multiple symptoms, signs, and clinical diagnostic data were common to the first 10 reported cases. Components of the case definition were selected from these cases, emphasizing quantitative signs that were the most objective and reproducible (weakness and decreased reflexes) and diagnostic tests that were available to most clinicians (CSF evaluation, neuroimaging including CT scan or MRI, and electrodiagnostic testing). Reported new pain and numbness were judged to be supportive of the syndrome but were not included in the case definition[4].

### Additional Case Finding

We conducted additional case-finding efforts at the local, regional, national, and international levels, using a multi-faceted approach. International classification of disease (ICD)-9 code searches for acute (357.0) and chronic inflammatory polyneuropathy (357.81), and idiopathic progressive polyneuritis (356.4) covering January 1, 2002 to November 30, 2007 were performed at area hospitals. Medical records were reviewed for work history and clinical-case criteria. Lists of all persons undergoing electrodiagnostic tests (nerve conduction studies or electromyography) from 1997 through 2007 were cross-matched with a list of all Plant A workers employed during the same period. MDH issued health alerts and press releases requesting reports of potential cases. Neurologists near regional U.S. pork-processing abattoirs were contacted directly. Active case finding continued through December 2009 via regular contact with onsite Plant A medical staff and neurologists in the community.

Domestic animal health agencies at both the state and federal level were contacted to assist in the investigation. All 26 U.S. federally inspected swine abattoirs with >500 employees were initially surveyed to identify abattoirs with similar brain removal techniques. Site visits were conducted at those abattoirs with similar techniques; occupational health and medical records were reviewed; and local primary-care physicians and regional neurologists were queried. Suspect cases, former employees, and head-table workers were interviewed by using a questionnaire modified from the Minnesota case-control study form. The World Organisation for Animal Health (OIE) was contacted to identify member countries using the similar techniques. Additional case reports were sought nationally through postings on the American Academy of Neurology and the Centers for Disease Control and Prevention Epi-X websites, and Emerging Infections Network electronic mailings. International contacts were made also to the Health Canada, the World Health Organization (WHO), and United States-Mexico Border Health Commission (USMBHC) to seek additional case reports.

### Case-Control Study

A case-control study was conducted at Plant A from December 4–11, 2007. Confirmed and probable cases included all employed workers with new onset of neurologic illness not explained by another cause, and findings meeting clinical and diagnostic criteria (Figure 1). Two unmatched control groups of employed Plant A workers were used — a random sample of warm-room workers (including head-table workers) and all head-table workers not meeting the case definition. Among those who were randomly identified, all provided written consent in their language of their choice. All subjects were interviewed in English or Spanish based on the subject's language choice with an extensive, standard questionnaire modified from the initial case questionnaire. Controls reporting mild neurologic symptoms (e.g., numbness, tingling, or weakness consistent with carpal tunnel entrapment syndrome) within the past year, but not meeting the case definition criteria, were excluded given the known higher prevalence of repetitive motion associated neurologic symptoms among workers in similar occupations[5].

Univariate and multivariate analyses were conducted to identify predefined potential risk factors included in the standardized questionnaire. Statistical analysis was completed with SAS software, version 9.1 (SAS, Cary, NC). Univariate analysis was performed with *X^2^* tests (two-tailed) or Wilcoxon-Mann Whitney tests. Statistically significant variables (P<0.05) by univariate analysis were entered into multivariate logistic regression models, along with sex and age.

### Laboratory Testing

Sera and throat specimens were collected from all consenting cases and controls. Cases and controls could consent to the collection of sera and throat independently. Throat swabs were pooled into 24 aliquots and plated in embryonated chicken eggs and on 10 viral culture cell lines: BT (bovine turbinate), PK15 [porcine kidney (ATCC® Number: CCL-33™)], BHK (baby hamster kidney), PPK (primary porcine kidney), MDCK (Madine-Darby canine kidney), CRFK (Crandell-Reese feline kidney), PAM (porcine alveolar macrophages), MARC-145 (monkey kidney), and Vero (monkey kidney). All supernatants were tested for hemagglutination and examined by transmission electron microscopy for viral elements. Indirect immunofluorescence was performed for encephalomyocarditis virus, hepatitis E virus (HEV), transmissible gastroenteritis virus, porcine adenovirus, porcine rotavirus, porcine reovirus, swine influenza, porcine teschovirus, porcine enterovirus A, pseudorabies virus, and porcine parvovirus.

A second throat swab was inoculated on Campylobacter selective media. As part of the case-control study, rectal swabs were not requested because of concern that this would decrease the participation rate. Isolation was performed on Campylobacter plates.

Among consenting cases with recent onset of symptoms stools were collected and tested for pathogens as part of the Unexplained Diarrhea Project, MDH employing 27 different pathogen specific testing modalities including PCR, culture, and antigen detection[6]. Additionally, broad range16S PCR was performed on blood specimens from the four most recent cases using previously published primers[7].

Serum levels of cytokines and chemokines [tumor necrosis factor α (TNFα), interferon γ (IFNγ), interleukin 1β (IL1β), interleukin 2 (IL2), interleukin 4 (IL4), interleukin 6 (IL6), interleukin 8 (IL8), interleukin 10 (IL10), interleukin 12p40 (IL12p40), monocyte chemotactic protein 1 (MCP1), IFNγ-inducible protein 10 (IP10), Macrophage inflammatory protein 1β (MIP1β)] in 15 cases and 53 control samples from abattoirs with cases were analyzed in triplicate using a multiplexed, bead-based cytokine immunoassay (Upstate Beadlyte® human multi-cytokine/chemokine kit, Millipore, St. Charles, Missouri) and the Luminex^100^ detection system (Luminex Corporation, Austin, Texas)[8], according to the manufacturer's protocols. Data were analyzed with StatView for Windows, version 5.0.1 (SAS Institute, Cary, NC). Two-tailed nonparametric Mann-Whitney U tests were employed for non-Gaussian distributions and *X^2^* tests were used to analyze categorical data.

Cytokine and chemokine data from all subjects (ill exposed, exposed non-ill, non-exposed non-ill) were additionally submitted to a principal components analysis (PCA), an exploratory method transforming an original set of variables into a smaller set of uncorrelated variables (factors or components), and summarizing most of the information of the original variables.[9] Variables were log transformed (log10[*x* + 1]) before PCA to reduce skewness. Extracted components were rotated orthogonally and obliquely, and component extraction was guided by the Kaiser criterion and scree test. Items with loadings on components ≥0.4 were retained. Additional criteria requiring significance for Bartlett's test of sphericity (P<0.05) and Kaiser-Meyer-Olkin statistic value >0.7 were applied. A three-factor, oblique solution best explained the data. Two-tailed nonparametric Kruskal-Wallis (ill exposed vs. exposed non-ill, vs. non-exposed, non-ill group comparisons) and Mann-Whitney U tests (ill exposed vs. both subsets of controls) were employed to compare factor scores, as distributions deviated from normality.

## Results

### Case Finding and Case Characteristics

**Figure 2:**
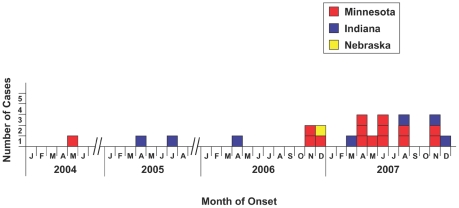
Minnesota, Indiana, and Nebraska immune-mediated polyradiculoneuropathy cases by month of illness onset and state.

**Figure 3:**
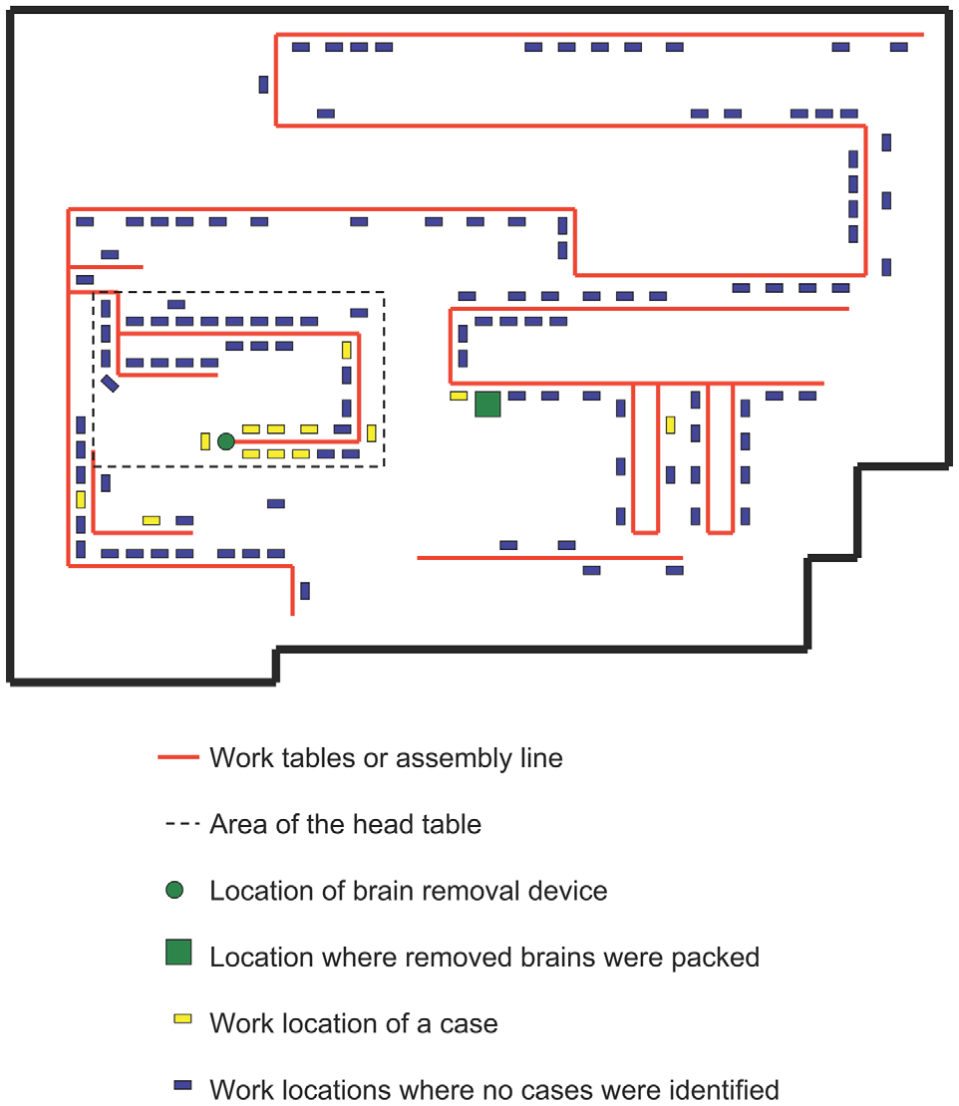
Schematic of warm-room processing area. Schematic represents the work stations on an assembly line within the warm-room of Plant A for the 13 cases that were able to be contacted. All cases reported working at or frequently visiting the head-table or exposure to brain tissue in Plant A.

Fifteen confirmed IP cases were identified in Minnesota, all were Plant A workers. All cases were identified by Plant A nursing staff and the two reporting medical centers. Eleven cases presented with symptoms in 2007, three in 2006, and one in 2004 (Figure 2). Eight (53%) were female; median age was 34 years (range, 21–54 years); and 80% were Hispanic. Per Plant A management, age and ethnicity of cases were similar to that in the abattoir as a whole; however, the proportion of female cases (53%) was higher than Plant A female workers overall (reported as 25%). Time of employment of cases at Plant A ranged from 3 months to 21 years (median, 13 months). All cases worked in the warm-room, and nine worked at the head-table (Figure 3). All cases reported working at or frequently visiting the head-table, or exposure to brain material in Plant A.

All had no other evident alternative diagnosis to explain symptoms following evaluation for various metabolic, inherited, para- or post-infectious, or neoplastic neuropathies[4]. All had limb numbness, decreased strength, and hypo- or areflexia; 13 (87%) described “tingling” in the limbs. Fifteen (100%) had at least one abnormality on electrodiagnostic testing consistent with axonal or demyelinating peripheral neuropathic features in affected limbs which could not be attributed to an underlying chronic disease process. Of 11 patients with spinal cord magnetic resonance imaging (MRI), 10 (91%) had nerve root enhancement and one had longitudinal intramedullary gray matter swelling. Eleven cases (73%) had CSF testing performed; the median CSF protein was 155 mg/dL (range 75–231; normal, <45 mg/dL) and median CSF white blood cell count (WBC) was 2 cells/mm^3^ (range 1–163; normal, <5 cells/mm3). Thirteen (87%) patients were tested for anti-nuclear antibody (ANA), and 9 (69%) were abnormal with the median of 1.7 units (range 0.5 to 7.1 units, (normal <1.0). Serum protein electrophoresis (SPEP) was performed in 10 with polyclonal hypergammaglobinemia in 4 (40%) and none with monogammaglobinema. Two cases were hospitalized, with lengths of stay of 3 and 42 days. Additional patients with neurologic symptoms including pure sensory features without muscle weakness, reflex change, or electrophysiological abnormalities required for our case definition were evaluated but did not meet the case definition criteria for this epidemiologic study[4].

Active case finding and passive reporting from clinicians and the public in Minnesota through December 2009 did not detect additional cases. No additional cases were identified from Plant A occupational or Workers' Compensation records. No reports of illness were received from other regional abattoirs. Fourteen of 15 cases were re-identified through one or more of the additional case finding efforts.

### Case-Control Study

Thirteen of 15 cases were current or recent Plant A workers and were able to be contacted; these workers were enrolled in the case-control study. Multiple unsuccessful attempts were made to contact the two additional cases. Controls included 49 warm-room workers and 56 head-table workers after those experiencing neurologic symptoms were excluded (four (8%) and three (5%) from the two groups, respectively). In the univariate analysis including warm-room controls (Table 1), cases were more likely than controls to have ever worked at the head-table (odds ratio [OR], 6.9; 95% confidence interval [CI], 1.8–26.6), to have ever removed skeletal muscle from the backs of swine heads, known commonly as backing heads (OR, 10.4; 95% CI, 1.7–65.8), and to have ever worked backing heads or removing brains (OR, 14.7; 95% CI, 2.4–89.1). Only one case and no controls ever had the job of removing brains, so this variable was combined with the backing heads variable for analysis. When comparing cases who worked at the head-table (n = 9) with non-ill head-table-worker controls (n = 56), ever backing heads (OR, 6.7; 95% CI, 1.4–31.8) and ever removing brains or backing heads (OR, 7.5; 95% CI, 1.7–34.0) were associated with illness (Table 1). Median ages for head-table cases and controls were 37 and 29 years, respectively (P = 0.01). Prior vaccinations, medications, travel, work elsewhere, time working at Plant A, and exposure to cleaning chemicals, insecticides, pesticides or other animals were not associated with illness using either control group.

**Table 1:** Univariate analysis and multivariate analysis of risk factors for immune-mediated polyradiculoneuropathy.

**Univariate and Multivariate Analysis Using Warm-Room Controls**						
**Potential risk factor**	**Cases**	**Controls**	**OR (95% CI)**	***P*** ** value** a	**AOR** b **(95% CI)**	***P*** ** value** a
Female, no. (%)	7 (54%)	12 (24%)	3.6 (0.9–15.5)	0.09	2.7 (0.6–13.0)	0.19
Age, years						
Median	32	27		0.23		0.37
(range)	(21–51)	(18–59)				
Ever worked at head-table, no. (%)	9 (69%)	12 (24%)	6.9 (1.8–26.6)	0.006	6.6 (1.6–26.7)	0.008
cEver backed heads, no. (%)	4 (31%)	2 (4%)	10.4 (1.7–65.8)	0.01	6.3 (0.8–47.1)	0.07
Ever Backed heads/Removed brains, no. (%)	5 (38%)	2 (4%)	14.7 (2.4–89.1)	0.003	10.3 (1.5–68.5)	0.01
Median distance from brain operation, m (ft)	5.8 (19.1)	13.8 (45.2)		0.04		0.14
Median minimum distance from brain	3.9 (12.9)	7.6 (24.8)		0.01		0.07
operation, m (ft)						
d≤3.1 m (10 ft) from brain operation, no. (%)	5 (38%)	2 (5%)	17.5 (2.5–122.5)	0.004	9.9 (1.2–80.0)	0.03
d3.2 to 6.1 m (11 to 20 ft) from brain operation,	4 (29%)	10 (25%)	2.8 (0.6–13.4)	0.20	2.7 (0.5–13.4)	0.20
no. (%)						
d≥6.2 m (20 ft) from brain operation, no. (%)	4 (29%)	28 (70%)	Referent		Referent	
Total time at Plant A, months						
Median	18.3	15		0.84		
(range)	(3–251)	(2–190)				
**Univariate and Multivariate Analysis Using Head-Table Controls**						
**Potential risk factor**	**Cases**	**Controls**	**OR (95% CI)**	***P*** ** value** a	**AOR** b **(95% CI)**	***P*** ** value** a
Female, no. (%)	4 (44%)	13 (23%)	2.7 (0.5–13.9)	0.23	1.3 (0.19–8.8)	0.80
Age, years						
Median	37	29		0.01		0.01
(range)	(27–51)	(19–61)				
cEver Backed heads, no. (%)	4 (44%)	6 (11%)	6.7 (1.4–31.8)	0.03	7.7 (1.1–53.0)	0.04
Ever Backed heads/Removed brains, no. (%)	5 (56%)	8 (14%)	7.5 (1.7–34.0)	0.01	13.5 (1.9–96.2)	0.009
Median distance from brain operation, feet	14.7	20.2		0.04		0.20
Median minimum distance from brain	6.7	14.89		0.01		0.01
operation, feet						
d≤3.1 m_(10 ft) from brain operation, no. (%)^e^	5 (56%)	8 (15%)	7.2 (1.6–32.7)	0.01	12.7 (1.8–91.4)	0.007
d>3.1 m (10 ft) from brain operation, no. (%)^e^	4 (40%)	46 (85%)	Referent		Referent	
Total time at Plant A, months						
Median	18.8	18		0.76		
(range)	(3–251)	(1–203)				

Abbreviations: CI, confidence interval; OR, odds ratio; AOR, adjusted odds ratio

a *P* value of<0.05 was considered statistically significant. All probabilities are two-tailed.

b Job and work location variables were entered into separate multivariate logistic-regression models along with sex and age. Multivariate results for sex and age (OR, 95% CI and *P* values) taken from model that included work location.

c Removing skeletal muscle from the back of hog heads.

d Some employees reported multiple job stations. Distance categories were created using the distance of the closest reported job station from the brain removal operation.

e All workers at the head-table were <6.2 m (20 ft) from the brain removal operation, so only two distance categories were used.

Distance from the employee-reported job station to the brain-removal operation was calculated. Some employees reported multiple job stations. When we compared the distance of the employee-reported job station closest to the brain-removal operation, the distances were significantly different for cases to warm-room controls. (Table 1). We separated minimum distance measurements into three categorical variables (≤3.1, 3.2–6.1, and ≥6.2 m); cases were more likely than warm-room controls to have worked ≤3.1 m from the brain-removal operation (OR, 17.5; 95% CI, 2.5–122.5). A similar association was identified for head-table cases, who more often worked within 0–3.1 m (versus 3.2–6.1 m) of the brain-removal operation, compared with head-table controls.

Colinearity of variables for minimum distance and specific job precluded comparison in the multivariate model. However, in separate models controlling for possible effects of age and sex, ever having worked at the head-table, ever having backed heads or removed brains, and having the closest job station located ≤3.1 m from the brain-removal operation were each more common among cases than warm-room controls (Table 1). Results were similar when comparing cases and controls who only worked at the head-table in multivariate models. Sex was not significantly associated with illness, and older age was only associated in models comparing head-table cases and controls.

### Environmental Assessment

Plant A processes hogs during two shifts, 5 or 6 days a week. Workers apply for specific jobs on the basis of seniority and ability and remain at those positions until successfully bidding for a new position. Safety equipment was consistent with industry standards, which does not include respiratory protection.

Plant A used approved chemicals and in approved concentrations. Maintenance records were unremarkable. Plant A had increased the line speed twice since 2003; from 1,200 to 1,300 hogs per hour in April 2003 and to 1,350 hogs per hour in 2006.

**Figure 4:**
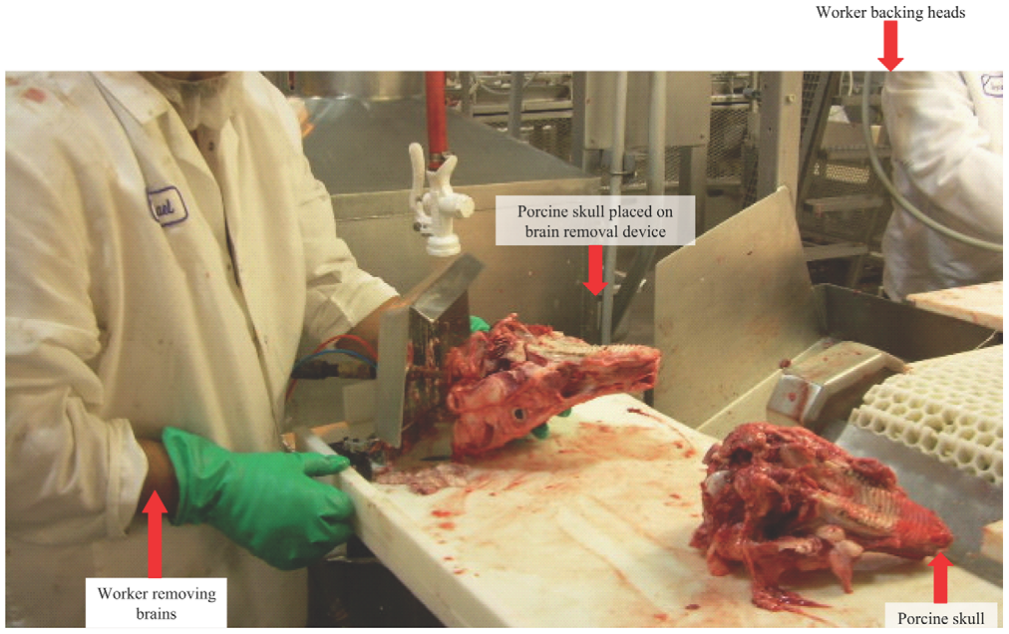
Photograph of brain removal compressed-air device during operation.

Inspection of Plant A revealed that since October 1998, a compressed-air device was used to harvest brains from detached heads (Figure 4). The immobile compressed-air device was located at the last station of the head-table. It consisted of a stainless steel tube connected to a compressed-air line mounted on a plastic table, and welded to stainless steel plates. The operator placed the swine head over the tube through the foramen magnum. This action tripped a wire air trigger mechanism. The compressed-air, pressurized to 90 psi, liquefied and extruded brain material through the foramen magnum, creating splatter and aerosolized droplets of brain tissue. The brain material was collected in a large pail below the compressed-air device and transferred to another area in the warm-room where the brains were packaged in 10 pound boxes for shipping. Residual brain material was observed on the worker removing brains and on workers who backed heads (the job closest to the brain removal operation) (Figure 4).

Plant A voluntarily discontinued operation of the brain-removal device following the initial site visit. No additional cases with illness onset after removal of this device have been identified through December 2009 during 24 months of observation.

### Additional Abattoir Investigations and Case Finding

Twenty-six U.S. swine abattoirs employing >500 workers were surveyed. Nine reported removing brains, and only three used a compressed-air device — Plant A, Plant B in Indiana, and Plant C in Nebraska. The OIE reported no member countries using similar brain removal techniques to Plant A. No additional cases were identified through international contacts.

At Plant B, 112 head-table employees were interviewed during February 5–8, 2008. Six confirmed IP cases and one probable case were identified by patient interview, physician referral, and query of former employees. Four (57%) cases were female, the median age was 28 years (range, 28–42), and all were Hispanic. Age and ethnicity were reported to be representative of plant workers; however, the number of female cases (57%) was disproportionately higher than the female worker population (38% reported by plant management). Six of seven cases were hospitalized for IP-related symptoms, with a median hospital stay of 8.5 days (range, 5–14 days)[10]. Four cases developed symptoms in 2007, one in 2006, and two in 2005 (Figure 2).

Five cases and 106 controls were enrolled in a case-control study at Plant B. Cases were more likely to report having porcine brain tissue enter their eyes, nose, or mouth during work (OR, 12.8; 95% CI, 1.4–119.3) and investigators observed workers covered in brain material. Plant B employs 1,750 workers and slaughters 1,000 hogs/hour, an increase from 860 hogs/hour in February 2006. Plant B had been removing brains since 1993 using a compressed-air device. In contrast to Plant A, a floor foot pedal rather than a wire trigger, allowing for direct operator control, released the compressed air. Use of this device was discontinued after the investigation[10].

At Plant C, 67 head-table workers were interviewed during February 28–29, 2008; none met the case definition, and 43 reported working at the head-table. A query of three former workers who had terminated employment for medical reasons identified one worker who met the confirmed case definition. This worker removed brains using a compressed-air device which had a hand-held trigger mechanism allowing for greater operator control. Plant C slaughters hogs during one shift at 1,250/hour, increased from 1,200/hour in 2006. The abattoir had been removing brains with compressed air since 1998 and had been using the current design since 2005. Plant C discontinued the procedure during investigation of Plant A.

No common source of pigs between Plant A, B, and C was identified over the study period. The abattoirs were 400–800 miles apart; in addition, abattoirs in Minnesota and Indiana were identified that would be expected to have had a similar source of animals but did not use compressed air to remove brains. Cases were not identified in any of these other abattoirs.

An additional survey was conducted of the 121 front-line supervisors in the USDA Food Safety and Inspection Service to verify that no additional facilities were using or had recently used a compressed air harvest method. One hundred six of the 121 front-line supervisors with oversight of 621 U.S. swine abattoirs responded to the survey. None identified compressed air harvesting beyond the three plants identified (USDA, personal communication).

### Laboratory Testing

Testing was performed on throat swabs from all consenting participants in the Minnesota case-control study (n = 116) and pooled into 24 pools. Cytopathic effect was observed in a single pool. This pool had a nonspecific immunofluorescence staining pattern and herpes simplex virus was isolated. No virus was isolated from embryonated chicken egg inoculums. All supernatants were negative for hemagglutination and no viral elements were observed by transmission electron microscopy. Indirect immunofluorescence for all 11 target pathogens was negative. All Campylobacter throat cultures (n = 118) were negative.

16S rRNA PCR was negative on all four blood specimens tested. Stools from four Minnesota and two Indiana cases were tested for 27 different stool pathogens within median of 43 days (range 33–261 days) from clinical onset. No consistent organism was identified: one case *Campylobacter coli* and *Blastocystis hominis*, one case Adenovirus and Microsporidium, one case *Endolimax nana* cysts and *Microsporidium spp*., one case norovirus, one case *Shigella flexneri*, and one case *Microsporidium spp*.

**Figure 5:**
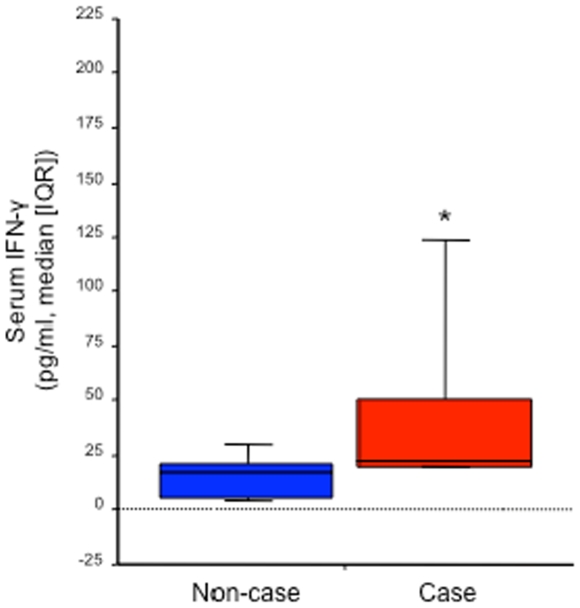
Serum IFNγ levels among swine abattoir workers experiencing immune mediated polyradiculoneuropathy versus non-ill workers.

Laboratory evaluation of case and control samples did not reveal evidence of a specific infectious etiology for IP (Table 2). Elevated serum levels of interferon-gamma (IFN-γ) were discovered among cases (median, 21.7 pg/ml; range, 6.2–199.8 pg/ml) versus controls (median, 14.8 pg/ml; range, 2.2–50.1 pg/ml) (P<0.001) (Figure 5). Approximately 94% of cases had IFN-γ values above the control median (P = 0.002).

**Table 2:** Serologic testing performed for human and swine infectious agents in the laboratory evaluation of immune- mediated polyradiculoneuropathy among cases and controls.

Infectious Agent^a^	Cases n (%)	Controls n (%)	OR (95% CI)
	**n = 13**	**n = 81**	
Swine influenza virus (H2) IgG +	4 (31%)	3 (4%)	b11.6 ( 2.2–60.1)
Encephalomyocarditis virus IgG +	0 (0%)	4 (5%)	
Porcine circovirus Type 2 IgG +	0 (0%)	0 (0%)	
Porcine enterovirus IgG subtype 1–8 +	0 (0%)	0 (0%)	
Porcine reproductive and respiratory	0 (0%)	0 (0%)	
syndrome (PRRS) virus IgG +			
Porcine hemagglutinating	2 (15%)	1 (1%)	b14.5 (1.2–174)
encephalomyelitis virus IgG +			
*Mycoplasma hyopneumoniae* IgG +	0 (0%)	0 (0%)	
Hepatitis E virus (HEV)^c^	**n = 15**	**n = 84**	
HEV IgG +	4 (27%)	17 (20%)	1.42 (0.41–5.1)
HEV IgM +	0 (0%)	0 (0%)	

Abbreviations: CI, confidence interval; OR, odds ratio.

a Blood specimens were collected on 102 consenting participants in the Minnesota case-control study. Not all cases or controls had adequate specimen for the complete battery of blood tests performed.

b Differences between cases and controls were noted in the serology results for swine influenza virus and porcine hemagglutinating encephalomyelitis virus; however, these results accounted for <32% of the cases.

c Serum was evaluated by commercially available (MP Diagnostics) and in-house enzyme immunoassays using recombinant ORF-2 and ORF-3 proteins as antigens. Stools from three IP cases were evaluated for HEV RNA by RT PCR, of which all were negative.

**Figure 6:**
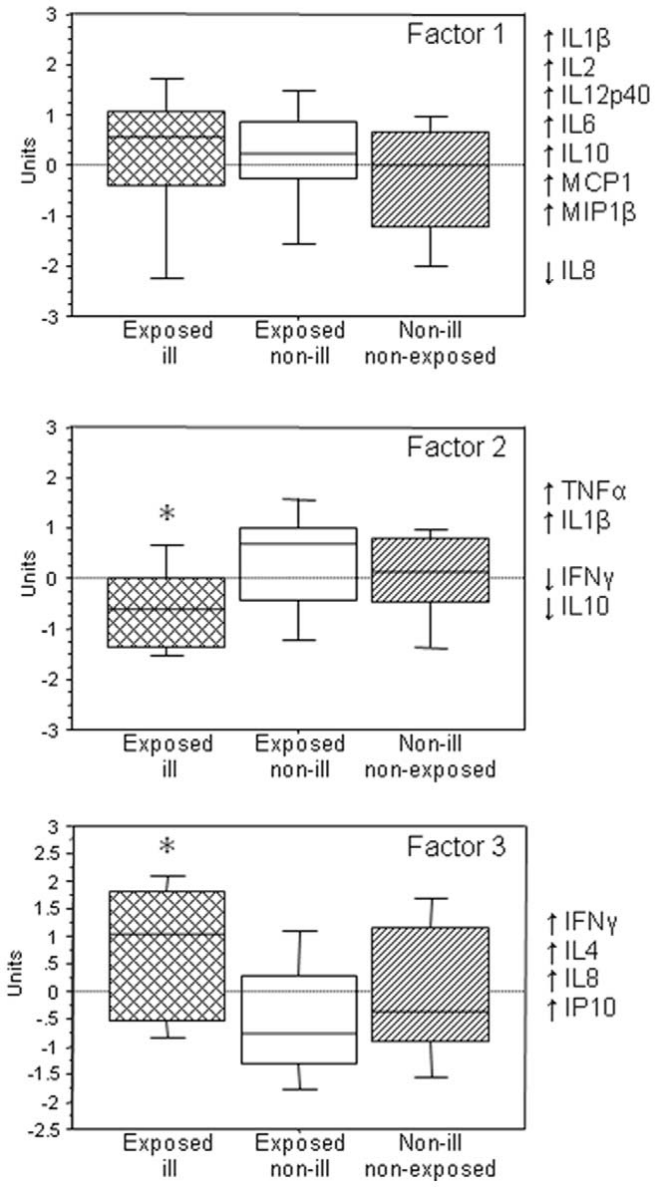
Two principal component analysis of ill, exposed non-ill, and non-exposed, non-ill workers. Two principal components explain nearly a third of the variance in cytokines and chemokine levels among swine abattoir workers experiencing immune-mediated polyradiculoneuropathy, exposed non-ill workers, and non-exposed, non-ill workers. Analysis includes cases and controls from the Minnesota and Indiana abattoir. Exposed ill individuals include 15 probable or confirmed cases. Exposed non-ill includes 25 unaffected individuals that worked at the headtable or in the headroom. Non-exposed non-ill includes 28 unaffected individuals that did not work at the headtable or in the headroom.

Exploratory principal components analysis yielded three groups of cytokines with increased or decreased levels referred to as factors, with eigenvalues >1, together accounting for 71.5% of the total common variance. The first factor (elevated IL1β, IL2, IL12p40, IL6, IL10, MCP1, and MIP1β; decreased IL8) accounted for 44.7% of the variance; factor 2 (elevated TNFα, IL1β; decreased IFNγ, IL10) accounted for 15.0% of the variance, and factor 3 (elevated IFNγ, IL4, IL8, and IP10) accounted for 11.8%. Emerged factor scores for factor 2 were lowest among the ill exposed subjects (median, −0.61; range, −2.59 to 2.53) and highest for the exposed non-ill controls (median 0.69; range, −1.41 to 2.21), with non-exposed, non-ill subjects falling in between the two exposed groups (median, 0.12; range, −1.83 to 1.08) (P<0.01). Factor 3 scores were highest among the ill exposed group (median, 1.05; range, −0.97 to 2.57), followed by the non-exposed non-ill controls (median, −0.37; range, −1.94 to 2.42) and then the exposed, non-ill control subjects (median, −0.74; range, −2.03 to 2.78) (P<0.01) (Figure 6). Similarly transformed IFNγ levels were highest among ill, exposed subjects (median, 1.35 pg/ml; range, 0.86 to 2.30 pg/ml), followed by levels among non-exposed, non-ill controls (median, 1.24 pg /ml; range, 0.53 to 1.68 pg/ml), and lowest among exposed, non-ill subjects (median, .91 pg/ml; range, 0.50 to 1.71 pg/ml) (P = 0.002). Proinflammatory cytokines (elevated IFNγ, IL4, IL8, and IL10) were significantly higher in cases compared to brain-exposed and non-exposed controls. Brain-exposed controls had lower proinflammatory cytokines compared to non-exposed controls.

## Discussion

We report the occurrence of a novel immune-mediated polyradiculoneuropathy in swine abattoir workers. We identified 15 cases in Minnesota, seven in Indiana, and one in Nebraska (Figure 2). These cases were defined primarily by the prominence of early sensory and motor neurologic symptoms, easily identified motor and reflex examination deficits on neurological examination, and specific electrophysiological findings. Although additional workers presented with neurologic symptoms, the observed symptoms were purely sensory and did not meet the epidemiologic case definition.

The case definition was developed at the onset of the investigation for the purpose of identifying risk factors associated with the illness. For this purpose, in the absence of a clear biologic marker, inclusion of only those individuals with the most reproducible signs and readily available diagnostics was important. All cases had some degree of sensory symptoms[4], however if those with only sensory symptoms were included in the case definition, this would have increased the likelihood of misclassification bias where persons with sensory symptoms due to another cause would be included, given the high frequency of factors such as repetitive motion injury, prolonged standing, and persistently cold conditions in this occupational setting[11]. Similarly, excluding persons with mild neurologic symptoms that did not meet the case definition decreased the chance of misclassification bias within the controls. Excluding the mild or asymptomatic cases of disease is a common practice in epidemiologic studies and outbreak investigations.

The epidemiologic features of the cases are distinguished by association with exposure to porcine brain harvested by compressed air. Although onsite investigations were not conducted at abattoirs that removed brains whole or did not remove brains, none of those abattoirs reported knowledge of unusual neurologic illness among their employees. All cases in the implicated abattoirs reported porcine brain exposure. In the Minnesota abattoir, working at the brain-removal job or the job closest to it (backing heads), or working at a distance within 3.1 meters from the brain-removal operation, was associated with illness. Jobs of backing heads and removing brain at the head-table are considered preferred jobs. Workers with more seniority successfully bid for these jobs, possibly explaining why age was significant when analyzing by head-table controls. No additional cases have been identified with onset dates after the process ceased in any of the implicated abattoirs.

Autoimmunity appears to be the likely pathogenic mechanism of IP induced by exposure to aerosolized porcine brain matter. Mucous membrane contact, inhalation, or possibly through contact with broken skin are the most likely routes of entry based on the association of cases with the brain-removal operation, our observations of the mist of brain created by the removal process, and the presence of residual brain material on nearby workers. Exposure to neural tissue including sheep brain[12], [13], [14], [15], [16], peripheral nerve myelin[17], [18], [19] and bovine gangliosides[12], [13], [14], [15] has been previously epidemiologically linked with development of autoimmune neuropathy. While similar illnesses have been described following injection of neural proteins, to the authors' knowledge this is the first time that a respiratory, mucosal, or through broken skin exposure is the likely route of entry as we have implicated. We cannot exclude the possibility that the pathogenesis of IP involves either an infection or direct toxic effect related to a component of porcine brain. However, no toxic or infectious cause was identified despite a comprehensive exposure history interview, review of chemicals used in the abattoirs, and extensive laboratory testing for infectious agents.

An autoimmune mechanism in IP is supported by higher levels of IFNγ in cases than in controls, as elevated IFNγ has been observed among persons experiencing acute or chronic inflammatory demyelinating polyradiculoneuropathies (AIDP or CIDP)[20], [21]. Further support for the importance of IFNγ-triggered cytokine cascades in IP derives from the parallel elevations of IFNγ and IFNγ-inducible protein of 10 kDa (IP-10) along with the Th2 cytokine, IL-4, and the chemokine, IL-8 (Figure 6). IP-10 is known to be elevated in the CSF of patients with inflammatory neuropathies and in inflamed peripheral nervous system the distribution of IP-10 mirrors that of the chemokine receptor CXCR3, its cognate receptor[22]. Intriguingly, control subjects who were exposed to brain material but did not manifest illness were revealed by factor analysis to deviate the most from the exposed ill group in their cytokine and chemokine profiles relative to non-exposed, non-ill controls. In comparison with ill exposed subjects, the factor 2 and 3 scores of exposed non-ill individuals suggested greater proinflammatory drive (increased TNFα, IL1β); less skew toward Th2-type cytokines (decreased IL4, IL10); and decreased IFNγ production. It is possible that diminished IFNγ production in the face of exposure to brain material protected exposed controls from developing disease. Regardless, among those exposed to brain tissue in this way reduced IFNγ secretion may have utility as a marker for disease severity.

Spontaneous secretion of IFNγ by peripheral blood mononuclear cells is increased at the peak of clinical disease among patients in whom AIDP is diagnosed and decreases during recovery in parallel with rises in serum concentrations of neutralizing IgG autoantibodies to IFNγ[23]. Additionally, IFNγ knock-out mice are protected from development of AIDP-like illness, implicating IFNγ as a critical component in development of autoimmune inflammatory neuropathies[24].

Changes in slaughter operations in the affected abattoirs might have affected the number of workers exposed to porcine brain or the intensity of exposure and could explain why illness occurred recently despite the fact that brain removal was occurring years prior. Workers from two abattoir reported being less efficient at removing brains after the line speed increased. These workers reported being unable to place the skulls completely on the brain removal device before triggering the compressed air, causing greater splatter of brain material. Plants B and C slaughtered fewer hogs per hour, and their compressed-air brain removal designs allowed for more control by the operator, potentially resulting in less brain splatter and fewer cases than in Plant A.

Proximity to brain removal was the strongest predictor of disease. However, certain workers positioned close to the brain-removal operation did not experience disease, indicating a potential role for genetic susceptibility or other host factors. This is supported by evidence among Semple rabies vaccine recipients where unique MHC class II alleles were identified among persons in whom neuropathy developed, compared with those who did not become ill[25].

One potential limitation of this investigation is the possibility that despite intensive case-finding efforts employing multiple methods, other IP cases were not identified. The workers in these abattoirs were reported to be highly mobile, often terminating employment rather than taking medical leave. As there was no specific biological marker for IP and symptoms were nonspecific, potential cases could have been overlooked. All interview data, including work history, were self-reported and were subject to recall bias. Although cases were identified among former abattoir workers, controls only included the workers who were working at the abattoir at the time of the investigation.

In summary, IP was more likely to occur among workers who reported close contact with brains or the job of removing brains by using compressed air. No additional cases have been identified with onsets after cessation of brain harvesting by compressed-air methods in Plants A, B, and C. Our findings indicate that swine abattoirs and other animal commodity abattoirs should not use compressed air to remove brains and should avoid any procedures that aerosolize CNS tissue. This outbreak highlights the potential for respiratory or mucosal exposure to cause an immune-mediated illness in an occupational setting and the importance of health care providers taking a careful work place exposure history. The cooperation between human and animal health organizations provided an optimal framework for this investigation and demonstrates the synergy needed to address emerging issues at the human-animal interface.

**Note:** The findings and conclusions in this report are those of the author(s) and do not necessarily represent the views of the Centers for Disease Control and Prevention.

## References

[1] Centers for Disease Control and Prevention (2008). Investigation of progressive inflammatory neuropathy among swine slaughterhouse workers--Minnesota, 2007–2008.. MMWR Morb Mortal Wkly Rep.

[2] State of Minnesota Office of the Revisor of Statutes (2009). Minnesota Statute 144.05, General Duties of Commissioner. State of Minnesota Office of the Revisor of Statutes website. Accessed: January 2010.. https://www.revisor.mn.gov/statutes/?id=144.05.

[3] State of Minnesota Office of the Revisor of Statutes (2008). Minnesota Administrative Rules, Chapter 4605 Communicable Diseases. State of Minnesota Office of the Revisor of Statutes website. Accessed: January, 2010.. https://www.revisor.mn.gov/rules/?id=4605.

[4] Lachance DH, Lennon VA, Pittock SJ, Tracy JA, Krecke KN (2010). An outbreak of neurological autoimmunity with polyradiculoneuropathy in workers exposed to aerosolised porcine neural tissue: a descriptive study.. The Lancet Neurology.

[5] Yves R, Catherine H, Guillaume N, Marie-Christine P-C, Camille M (2008). Attributable risk of carpal tunnel syndrome according to industry and occupation in a general population.. Arthritis Care & Research.

[6] Fuller C, Cebelinski E, Bartkus J, Juni B, Smith K, Besser J (2008). Enhanced laboratory testing of enteric disease outbreaks of unknown etiology in Minnesota. 6th International Conference on Emerging Infectious Diseases.. Atlanta, GA.

[7] Weisburg WG, Barns SM, Pelletier DA, Lane DJ (1991). 16S ribosomal DNA amplification for phylogenetic study.. J Bacteriol.

[8] Prabhakar U, Eirikis E, Davis HM (2002). Simultaneous quantification of proinflammatory cytokines in human plasma using the LabMAP assay.. J Immunol Methods.

[9] Genser B, Cooper PJ, Yazdanbakhsh M, Barreto ML, Rodrigues LC (2007). A guide to modern statistical analysis of immunological data.. BMC Immunol.

[10] Adjemian JZ, Howell J, Holzbauer S, Harris J, Recuenco S (2009). A Clustering of Immune-mediated Polyradiculoneuropathy among Swine Abattoir Workers Exposed to Aerosolized Porcine Brains, Indiana, United States.. Int J Occup Environ Health.

[11] Dicker R, Gregg M (2008). Designing Studies in the Field.. Field Epidemiology. 3rd ed.

[12] Javier RS, Kunishita T, Koike F, Tabira T (1989). Semple rabies vaccine: presence of myelin basic protein and proteolipid protein and its activity in experimental allergic encephalomyelitis.. J Neurol Sci.

[13] Udawat H, Chaudhary HR, Goyal RK, Chaudhary VK, Mathur R (2001). Guillain-Barre syndrome following antirabies semple vaccine--a report of six cases.. J Assoc Physicians India.

[14] Hemachudha T, Phanuphak P, Johnson RT, Griffin DE, Ratanavongsiri J (1987). Neurologic complications of Semple-type rabies vaccine: clinical and immunologic studies.. Neurology.

[15] Hemachudha T, Griffin DE, Chen WW, Johnson RT (1988). Immunologic studies of rabies vaccination-induced Guillain-Barre syndrome.. Neurology.

[16] O'Connor KC, Chitnis T, Griffin DE, Piyasirisilp S, Bar-Or A (2003). Myelin basic protein-reactive autoantibodies in the serum and cerebrospinal fluid of multiple sclerosis patients are characterized by low-affinity interactions.. J Neuroimmunol.

[17] Abramsky TO, Teitelbaum D, Arnon R (1977). Experimental allergic neuritis induced by a basic neuritogenic protein (P1L) of human peripheral nerve origin.. Eur J Immunol.

[18] Lisak RP, Behan PO (1975). Experimental autoimmune demyelinating diseases: experimental allergic encephalomyelitis and experimental allergic neuritis.. Biomedicine.

[19] Smith ME, Forno LS, Hofmann WW (1979). Experimental allergic neuritis in the Lewis rat.. J Neuropathol Exp Neurol.

[20] Csurhes PA, Sullivan AA, Green K, Pender MP, McCombe PA (2005). T cell reactivity to P0, P2, PMP-22, and myelin basic protein in patients with Guillain-Barre syndrome and chronic inflammatory demyelinating polyradiculoneuropathy.. J Neurol Neurosurg Psychiatry.

[21] Jander S, Stoll G (2001). Interleukin-18 is induced in acute inflammatory demyelinating polyneuropathy.. J Neuroimmunol.

[22] Kieseier BC, Tani M, Mahad D, Oka N, Ho T (2002). Chemokines and chemokine receptors in inflammatory demyelinating neuropathies: a central role for IP-10.. Brain.

[23] Elkarim RA, Dahle C, Mustafa M, Press R, Zou LP (1998). Recovery from Guillain-Barre syndrome is associated with increased levels of neutralizing autoantibodies to interferon-gamma.. Clin Immunol Immunopathol.

[24] Bour-Jordan H, Thompson HL, Bluestone JA (2005). Distinct Effector Mechanisms in the Development of Autoimmune Neuropathy versus Diabetes in Nonobese Diabetic Mice.. J Immunol.

[25] Piyasirisilp S, Schmeckpeper BJ, Chandanayingyong D, Hemachudha T, Griffin DE (1999). Association of HLA and T-cell receptor gene polymorphisms with Semple rabies vaccine-induced autoimmune encephalomyelitis.. Ann Neurol.

